# Precipitation-Based Encapsulation of Fibrinogen in Calcium Carbonate for Non-Compressible Hemorrhage Control

**DOI:** 10.3390/ph19060923

**Published:** 2026-06-11

**Authors:** Henry T. Peng, Tristan Bonnici, Catherine Tenn, Christian J. Kastrup, Andrew Beckett

**Affiliations:** 1 Defence Research and Development Canada, Toronto Research Centre, Toronto, ON M3K 2C9, Canada; 2 Defence Research and Development Canada, Suffield Research Centre, Medicine Hat, AB T1A 8K6, Canada; 3 Versiti Blood Research Institute, Milwaukee, WI 53226, USA; 4 Department of Surgery, Division of Trauma and Acute Care Surgery, Medical College of Wisconsin, Milwaukee, WI 53226, USA; 5 Department of Biochemistry, Medical College of Wisconsin, Milwaukee, WI 53226, USA; 6 Department of Biomedical Engineering, Medical College of Wisconsin, Milwaukee, WI 53226, USA; 7 Department of Pharmacology and Toxicology, Medical College of Wisconsin, Milwaukee, WI 53226, USA; 8 Michael Smith Laboratories, University of British Columbia, Vancouver, BC V6T 1Z4, Canada; 9 St. Michael’s Hospital, University of Toronto, Toronto, ON M5B 1W8, Canada; 10 Royal Canadian Medical Services, Ottawa, ON K1A 0K2, Canada

**Keywords:** calcium carbonate, fibrinogen encapsulation, hemostatic particles, non-compressible hemorrhage, self-propulsion, tranexamic acid

## Abstract

**Background**: Uncontrolled hemorrhage, especially at non-compressible sites, remains a major cause of preventable trauma deaths. This study reports the development of fibrinogen-loaded calcium carbonate (CaCO_3_) microparticles that combine hemostatic activity with self-propelling capability for targeted delivery against blood flow, with a focus on understanding formulation-dependent trade-offs among particle yield, protein loading, clotting performance, and transport behavior. **Methods**: Microparticles were synthesized via a precipitation method using different carbonate sources and characterized for yield, morphology, size, and fibrinogen encapsulation. Hemostatic function was assessed using rotational thromboelastometry (ROTEM) in fibrinogen-deficient plasma. Propulsion behavior was evaluated following exposure to protonated tranexamic acid (TXA^+^), which triggers CO_2_ generation. Particle size and encapsulation were examined by microscopy and fluorescence imaging. **Results**: The precipitation method produced spherical micrometer-sized particles, with fibrinogen inclusion reducing yield and particle size relative to unload controls. Fluorescence microscopy confirmed successful encapsulation. Encapsulation efficiency varied with formulation, with sodium carbonate-based particles showing higher relative fibrinogen loading. ROTEM analysis demonstrated that fibrinogen-loaded particles significantly improved clot formation, increasing maximum clot firmness compared to fibrinogen-free particles, although performance remained formulation-dependent. TXA^+^-triggered propulsion achieved maximum speeds up to 4.221 cm/s. Fibrinogen-loaded particles exhibited longer activation lag times than unloaded particles, indicating a trade-off between hemostatic functionality and propulsion kinetics. **Conclusions**: Fibrinogen-loaded CaCO_3_ microparticles exhibit both hemostatic activity and chemically triggered motion in vitro. The study identifies key formulation-dependent trade-offs between particle yield, fibrinogen loading, clotting performance, and propulsion behavior. While these findings support the feasibility of combining localization and clot stabilization mechanisms, further studies under physiologically relevant flow conditions and in vivo models are required to evaluate their potential for active delivery in non-compressible hemorrhage.

## 1. Introduction

Uncontrolled hemorrhage, particularly from non-compressible internal bleeding, remains a leading cause of preventable death in both military and civilian trauma [1,2,3]. While conventional methods such as compression, hemostatic dressings, and tourniquets are effective for superficial, junctional or extremity bleeding [4,5], they are inadequate for non-compressible, deep, irregular, or high-flow bleeding, which accounts for over 60% of preventable trauma deaths [6].

Hemorrhage also poses significant risks in surgical contexts, particularly in cardiovascular and hepatic procedures, where uncontrolled bleeding can lead to prolonged operative times, infection, delayed healing, organ failure, and maternal mortality during childbirth [7]. Traditional hemostatic techniques such as electrocautery, suturing, and stapling are often ineffective in complex, deep, or irregular wounds and may exacerbate tissue damage.

Recent developments in topical and injectable hemostatic agents have focused on enhancing clot formation through a combination of biological and material-based mechanisms, including platelet activation, fibrin generation, and antifibrinolytic activity [8,9,10,11,12,13]. However, many current materials rely on passive diffusion or external application and are susceptible to displacement by blood flow. This limitation is particularly evident for particulate and powder-based systems, which, despite their high surface area and potential for minimally invasive delivery, may be washed away before effective clot formation occurs. Furthermore, incomplete retention at the injury site can reduce efficacy while increasing the risk of undesirable systemic distribution.

However, many current agents require manual pressure and are limited in their ability to reach deep or irregular bleeding sites. For example, field-deployed dressings like Combat Gauze and ChitoGauze rely on compression [14], while newer devices like XStat offer pressure-independent control but lack biodegradability and must be removed post-use [15]. Hemostatic powders, though promising for internal and irregular wounds due to their small size and high surface area [16,17], are often displaced by pressurized blood flow, limiting their efficacy [18,19]. Additionally, some powders pose embolization risks if they dissolve or disperse into circulation [20].

To overcome these limitations, self-propelling particles have been developed as a strategy to improve localization and retention of hemostatic agents [18]. These particles, composed of calcium carbonate (CaCO_3_), fibrinogen or thrombin, and protonated tranexamic acid (TXA^+^), generate CO_2_ gas upon contact with blood, propelling procoagulants into bleeding sites [21,22]. TXA^+^ enhances clot stability by inhibiting fibrinolysis [23], while calcium ions may further support coagulation and mitigate trauma-induced coagulopathy [24]. Studies have shown that dressings loaded with these particles outperform Combat Gauze in swine models of severe bleeding, even without manual pressure [25,26,27]. Furthermore, a self-propelling formulation consisting of CaCO_3_ particles and TXA^+^ demonstrated greater efficacy in inhibiting fibrinolysis and promoting hemostasis than a non-propelling formulation composed of CaCO_3_ and non-protonated TXA, as shown in in vitro fibrinolysis assays and in murine and swine models of hemorrhage [27].

Fibrinogen is a particularly attractive component for such systems due to its central role in clot formation and its early depletion during trauma [28]. We previously demonstrated the efficacy of CaCO_3_ particles loaded with fibrinogen [22]. However, simple mixing methods may result in incomplete loading and poor delivery to bleeding sites. To address this, we developed CaCO_3_-encapsulated fibrinogen particles using a water–oil–water emulsion method, which enabled encapsulation but involved complex processing and introduced limitations in scalability, reproducibility, and control over particle formation [29]. In contrast, precipitation-based synthesis offers a simpler and potentially more scalable approach; however, its ability to achieve efficient fibrinogen loading while preserving both hemostatic function and TXA^+^-driven propulsion remains unclear.

The key knowledge gap addressed in this study is therefore to define how precipitation-based encapsulation influences these coupled properties and to determine whether formulation parameters can be tuned to balance competing requirements. Rather than introducing a new hemostatic mechanism, the primary contribution of this work lies in establishing a synthetically simplified and tunable preparation method and providing systematic insight into formulation-dependent trade-offs between biological activity and transport behavior.

Specifically, we synthesize fibrinogen-loaded CaCO_3_ microparticles using a precipitation method and characterize their yields, morphology, particle size, fibrinogen content, hemostatic efficacy, and TXA^+^-triggered self-propulsion. By systematically varying key parameters—including carbonate source, concentration, reaction time, and mixing conditions—we evaluate their effects on particle yield, encapsulation efficiency, clotting performance, and propulsion characteristics. These results provide a framework for understanding the interplay between formulation and function and inform the rational design of multifunctional hemostatic materials with potential relevance for the management of severe bleeding, including non-compressible hemorrhage.

## 2. Results

A total of 27 formulations of CaCO_3_ particles, both unloaded and loaded with fibrinogen, were prepared under varying precipitation conditions. Results are presented in five subsections: (i) particle yield, (ii) particle morphology and size, (iii) gel electrophoresis and fibrinogen content, (iv) hemostatic properties, and (v) propulsion behavior.

### 2.1. Particle Yield

As shown in Table 1, particle yields varied substantially across formulations, ranging from 11% (Pre AC Fib2× 30 min) to 85% (Pre 2×SC 2×CaCl_2_ NoFib 30 min). The presence of fibrinogen consistently reduced particle yield across all carbonate sources, likely due to interference with CaCO_3_ crystallization. For example, SC-based particles at 0.33 M showed a yield reduction from approximately 80% to 40–60% (e.g., 82% for Pre SC NoFib 30 min vs. 54% for Pre SC FibCO_3_R 30 min; 78% for Pre SC NoFib 2×Spd 4 h vs. 47% for Pre SC FibCO_3_R 2×Spd 4 h). When both SC and CaCl_2_ solutions contained fibrinogen, yields dropped further to 37% and 36% (Pre SC Fib2× 2 h and Pre SC Fib2× 30 min, respectively). Reducing fibrinogen concentration from 20 to 15 g/L increased yield from 46% to 64% (Pre SC FibCO_3_R 2 h vs. Pre SC Fib_low_CO_3_R 2 h).

Similar trends were observed with AC and SBC systems. For instance, yields decreased from 35% to 30% (Pre AC NoFib 2 h vs. Pre AC FibCO_3_R 2 h), and from 41% to 26% (Pre SBC NoFib 2 h vs. Pre SBC FibCO_3_R 2 h). Doubling SBC concentration improved yield from 26% to 51% (Pre SBC FibCO_3_R 2 h vs. Pre 2×SBC FibCO_3_R 2 h). When both carbonate and calcium solutions contained fibrinogen, yields dropped further (e.g., 30% to 19% in Pre AC FibCO_3_R 2 h vs. Pre AC Fib2× 2 h).

Under identical conditions (1.65 mmol carbonate and calcium, 200 RPM, 2 h), SC produced the highest yield (46%), followed by AC (30%) and SBC (26%). Doubling CaCl_2_ concentration increased yield to 66%, and doubling both SC and CaCl_2_ further increased it to 73%. Conversely, reducing either CaCl_2_ or both SC and CaCl_2_ decreased yield to 39% and 29%, respectively. Variations in mixing speed (200 vs. 400 RPM) and mixing order had minimal impact, with yields ranging from 47 to 55%. Extending reaction time from 30 min to 4 h did not improve yield.

In summary, particle yield is primarily governed by carbonate and calcium concentrations and is consistently reduced by fibrinogen incorporation.

### 2.2. Particle Morphology and Size

**Figure 1:**
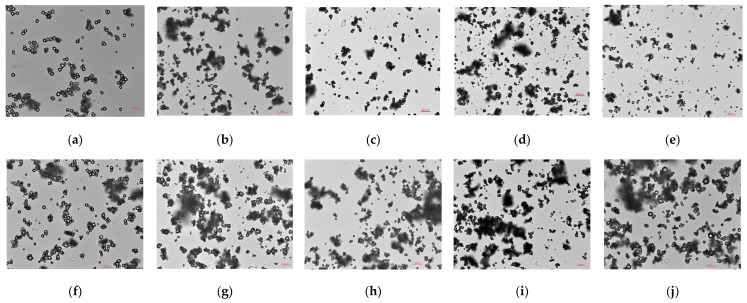
Light microscopy images of (**a**) Pre SC NoFib 30 min, (**b**) Pre SC FibCO_3_R 30 min, (**c**) Pre SC FibCO_3_R 2 h, (**d**) Pre SC FibCO_3_R 4 h, (**e**) Pre SC FibCO_3_R 2×Spd 30 min, (**f**) Pre SC FibCO_3_ 30 min, (**g**) Pre SC FibCl_2_ 30 min, (**h**) Pre SC FibCl_2_R 30 min, (**i**) Pre 2×SC 2×Cl FibCl_2_ 30 min, (**j**) Pre SC Fib2× 30 min. Particles were prepared by the precipitation method under various conditions. Scale bars represent 20 µm. See Table 1 and Section 4.2.1 for details of sample preparation and image acquisition.

**Figure A1:**
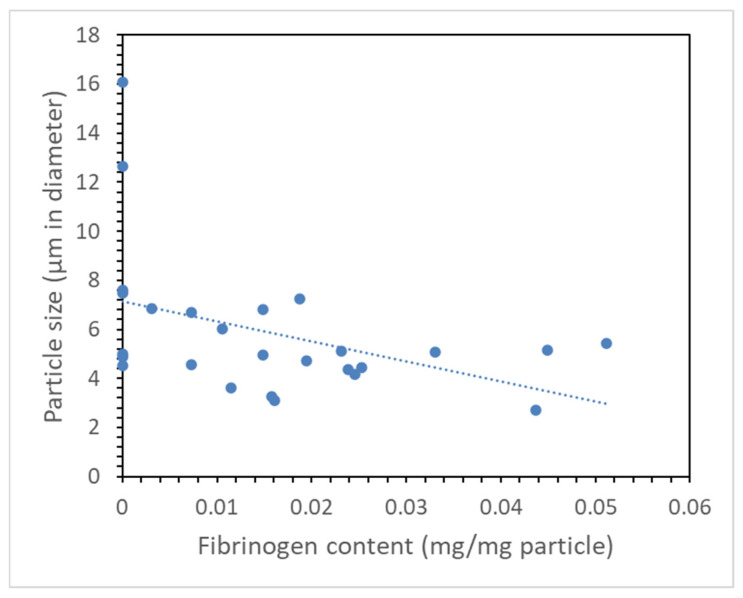
Effects of fibrinogen content on the size of CaCO_3_-encapsulated fibrinogen particles. A linear relationship with a Pearson correlation coefficient of −0.425 (*p* = 0.03) was observed.

Figure 1 shows light microscopy images of SC-based CaCO_3_ particles prepared under various conditions. All particles exhibited spherical morphology with bright central regions and darker edges, often forming aggregates. Fibrinogen inclusion generally reduced particle size and appeared to diminish crystallinity compared to NoFib controls. An inverse relationship between particle size and fibrinogen content was observed (Figure A1).

As summarized in Table 2, particle diameters ranged from 2.701 μm to 16.095 μm. The smallest particles were observed in Pre SC FibCO_3_R 2×Spd 30 min, and the largest in Pre AC NoFib 2 h. At equal molar ratios of SC and CaCl_2_, particles were smaller (Pre SC FibCO_3_R 2 h) than those prepared at lower or higher ratios (Pre SC FibCO_3_R 0.5×CaCl_2_ 2 h and Pre SC FibCO_3_R 2×CaCl_2_ 2 h). Fibrinogen-containing SC particles were consistently smaller than their NoFib counterparts (e.g., Pre SC Fib2× 30 min and Pre SC FibCO_3_R 30 min vs. Pre SC NoFib 30 min). Reducing fibrinogen concentration from 20 to 15 g/L increased particle size from 3.117 μm to 6.019 μm.

At the equal molar ratio and reaction time of 30 min, the mixing order and the solution containing fibrinogen also influenced size: adding SC to CaCl_2_ with fibrinogen produced the smallest particles (Pre SC FibCaCl_2_ 30 min), while the reverse produced the largest (Pre SC FibCO_3_ 30 min).

Doubling SC and CaCl_2_ concentrations increased particle size from 4.185 to 5.056 μm, while halving them reduced size from 5.634 to 4.716 μm. Increasing mixing speed from 200 to 400 RPM reduced particle size at 30 min (5.168 to 2.701 μm), but increased it at 4 h (4.347 to 6.684 μm). Notably, Pre AC NoFib 2 h and Pre SBC NoFib 2 h produced unusually large particles (>12 μm).

At equal molar concentrations, AC produced the largest particles, followed by SBC and SC (6.818 μm, 4.579 μm, and 3.117 μm, respectively). Similar to the SC-based particles, fibrinogen-containing AC and SBC particles were consistently smaller than their NoFib controls. When both AC and CaCl_2_ solutions contained fibrinogen, the particle size was even smaller. Doubling SBC concentration reduced particle size in NoFib samples (12.657 to 4.874 μm) but had minimal effect on fibrinogen-loaded particles.

**Figure 2:**
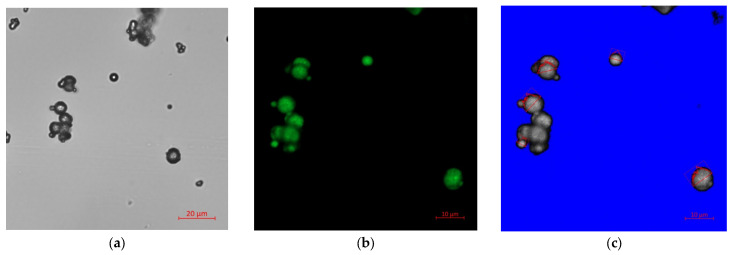
Light microscopy image (**a**) and fluorescence microscopy images of the particle Pre 2×SBC FibCO_3_R 2 h before (**b**) and after subtraction of its corresponding fibrinogen-free control (**c**). See Table 1 and Section 4.2.1 for details of sample preparation and image acquisition. Figure 2 (**c**) was previously published as Figure 2B in Ref. [30]. As indicated in (**c**) (highlighted in red), a path-dependent intensity profile was generated along the largest cross-section of the particles to visualize the spatial distribution of encapsulated fibrinogen across the particle diameter.

Fluorescence microscopy revealed a relatively uniform distribution of fibrinogen within the particles, with a concentrated core and a dark shell (Figure 2). This contrasts with light microscopy images, which primarily showed spherical particle morphology and aggregation patterns but did not provide insight into internal protein localization. The fluorescence images suggest that fibrinogen preferentially accumulates near the particle center during precipitation, forming a dense core. It should be noted that FITC labeling was used solely for visualization and may have influenced particle characteristics, including size and fibrinogen content.

In summary, fibrinogen incorporation reduces particle size and alters structure, but multiple interacting parameters govern morphology, and observed trends should be interpreted cautiously.

### 2.3. Gel Electrophoresis and Fibrinogen Content

**Figure 3:**
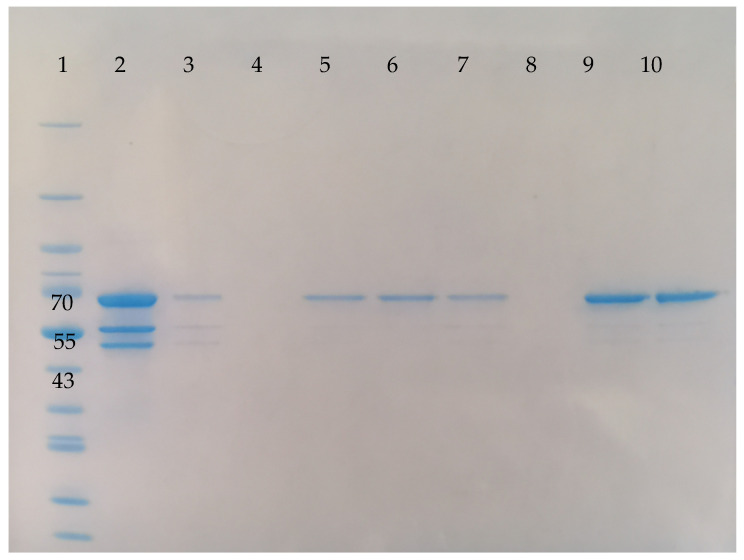
Coomassie-stained sodium dodecyl sulfate-polyacrylamide gel electrophoresis analysis of fibrinogen standards and CaCO_3_-encapsulated fibrinogen particle samples dissolved in TXA^+^ solution. Each sample except standard proteins (Mw STD) was reduced with 5% dithiothreitol and analyzed by gel electrophoresis. Indicated molecular weights were estimated by Mw STD with known molecular weights from 10 to 205 kDa. From left to right, each sample represents: Mw STD (Lane 1), fibrinogen standard (1 mg/mL, Lane 2), fibrinogen standard (0.2 mg/mL, Lane 3), fibrinogen standard (0.04 mg/mL, Lane 4), Pre 2×SBC FibCO_3_R 2 h (Lane 5), Pre SC FibCO_3_R 2 h (Lane 6), Pre SC 2×CaCl_2_ FibCO_3_R 2 h (Lane 7), Pre SC 0.5×CaCl_2_ FibCO_3_R 2 h (Lane 8), Pre SC FibCO_3_R 30 min (Lane 9), Pre SC FibCO_3_R 2×spd 30 min (Lane 10). See Table 1 for details of each batch of samples.

Gel electrophoresis was employed to detect and quantify fibrinogen encapsulated within CaCO_3_ particles. As shown in Figure 3, the fibrinogen standards exhibited the characteristic triplet of bands corresponding to the α-, β-, and γ-chains at approximately 64 kDa, 56 kDa, and 47 kDa, respectively (lanes 2 and 3) [31]. Band intensity decreased proportionally with fibrinogen concentration, ranging from 1 mg/mL to 0.04 mg/mL, confirming the sensitivity of the assay.

All particle samples exhibited a prominent α-chain band near 64 kDa, with varying intensities across formulations, indicating successful encapsulation of fibrinogen. Several samples also showed faint β- and γ-chain bands around 56 and 47 kDa, respectively, further confirming the presence of intact fibrinogen. Notably, the SC particle prepared with a lower CaCl_2_ concentration (Pre SC 0.5×CaCl_2_ FibCO_3_R 2 h, lane 8) showed no clear characteristic bands, indicating minimal fibrinogen encapsulation under those conditions. Fibrinogen content was estimated by comparing band intensities to those of the standards and is summarized in Table 2.

Fibrinogen content varied significantly across formulations. For SC-based particles, the sample prepared at an equal molar ratio of SC and CaCl_2_ (Pre SC FibCO_3_R 2 h) contained 0.0161 mg fibrinogen/mg particle, which was higher than those prepared at lower (0.0031 mg/mg, Pre SC 0.5×CaCl_2_ FibCO_3_R 2 h) or higher (0.0115 mg/mg, Pre SC 2×CaCl_2_ FibCO_3_R 2 h) calcium concentrations. When both carbonate and calcium solutions contained fibrinogen, encapsulation increased further (0.0231 mg/mg, Pre SC Fib2× 2 h).

Increasing reaction time from 30 min to 4 h did not increase the fibrinogen content in the Pre SC FibCO_3_R particles. In fact, the 30 min reaction (Pre SC FibCO_3_R 30 min) yielded 0.045 mg/mg, higher than the 2 h and 4 h reactions. This trend suggests that prolonged reaction times may lead to denature or diffusion of fibrinogen out of the forming particles, or reduced incorporation efficiency due to slower precipitation kinetics. As expected, increasing fibrinogen concentration from 15 to 20 g/L improved encapsulation (0.0105 vs. 0.0161 mg/mg).

Mixing order also influenced fibrinogen loading. Adding CaCl_2_ to fibrinogen-containing SC solution resulted in higher content (Pre SC FibCO_3_R 30 min), while doubling both carbonate and calcium concentrations increased fibrinogen content from 0.0149 to 0.0331 mg/mg (Pre SC FibCaCl_2_ 30 min vs. Pre 2×SC 2×CaCl_2_ FibCaCl_2_ 30 min). Doubling mixing speed had minimal effect at 30 min but reduced fibrinogen content at 4 h (from 0.0239 to 0.0073 mg/mg), further supporting the notion that extended exposure during precipitation may negatively affect fibrinogen retention. These findings highlight the importance of optimizing reaction time and mixing speed to balance particle formation and protein encapsulation efficiency.

Among carbonate sources, SC yielded the highest fibrinogen content (0.0161 mg/mg), followed by AC (0.0148 mg/mg) and SBC (0.0073 mg/mg). Doubling SBC concentration significantly improved encapsulation (to 0.0253 mg/mg, Pre 2×SBC FibCO_3_R 2 h).

It is noteworthy that formulation conditions may have opposing effects on yield and fibrinogen content. For example, while increasing CaCl_2_ concentration improved particle yield, the highest fibrinogen encapsulation was achieved at equal molar ratios of SC and CaCl_2_.

In summary, fibrinogen loading is highly formulation-dependent and often inversely related to yield, highlighting a key trade-off between production efficiency and protein incorporation.

### 2.4. Hemostatic Properties

To confirm the presence of fibrinogen within the particles and assess their hemostatic functionality, ROTEM assays were performed using human plasma with abnormally low fibrinogen levels and human whole blood obtained from healthy donors. Changes in viscoelastic properties were evaluated by CT, reflecting the onset of coagulation, and MCF, reflecting final clot strength, upon exposure to the particles.

As summarized in Table 2, all fibrinogen–CaCO_3_ particles enhanced hemostasis, evidenced by shortened CT and increased MCF compared to their corresponding control particles prepared without fibrinogen. CT values ranged from 473 to 1874 s, while MCF values ranged from 4 to 8 mm, with the highest MCF (8 mm) observed for Pre AC Fib2× 2 h. Preparation conditions, except for carbonate type, did not markedly influence hemostatic performance. Particles produced with AC and SBC exhibited greater effects (MCF: 7–8 mm) than those from SC (MCF: 4–6 mm), despite their relatively low fibrinogen content. No detectable coagulation occurred with any control particles, confirming the necessity of fibrinogen for clot formation.

**Figure A2:**
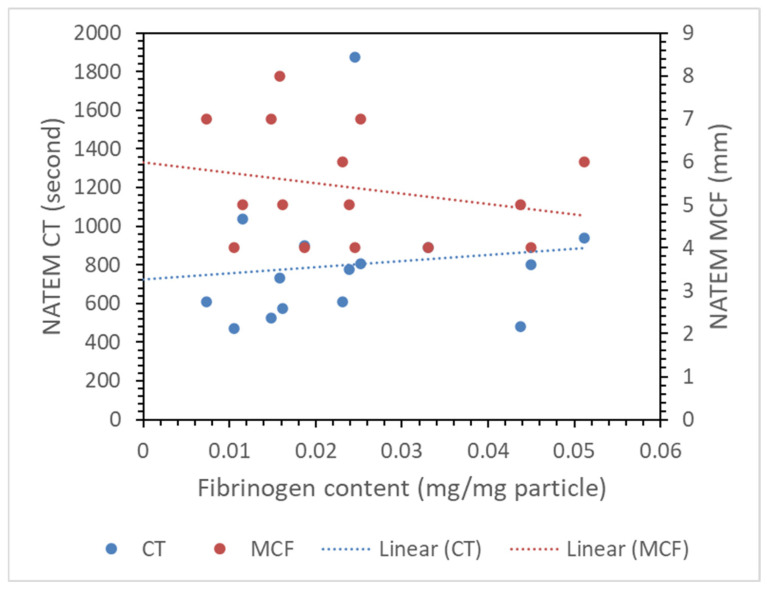
Effects of fibrinogen content on ROTEM clotting time (CT) and maximum clot firmness (MCF). ROTEM NATEM tests were performed with human plasma containing an abnormally low level of fibrinogen. It should be noted that all control particles prepared in the absence of fibrinogen resulted in non-detectable coagulation and were omitted. The Pearson correlation coefficient between the fibrinogen content and NATEM CT: r = 0.125 (*p* = 0.66), and NATEM MCF: r = −0.242 (*p* = 0.39).

**Figure A3:**
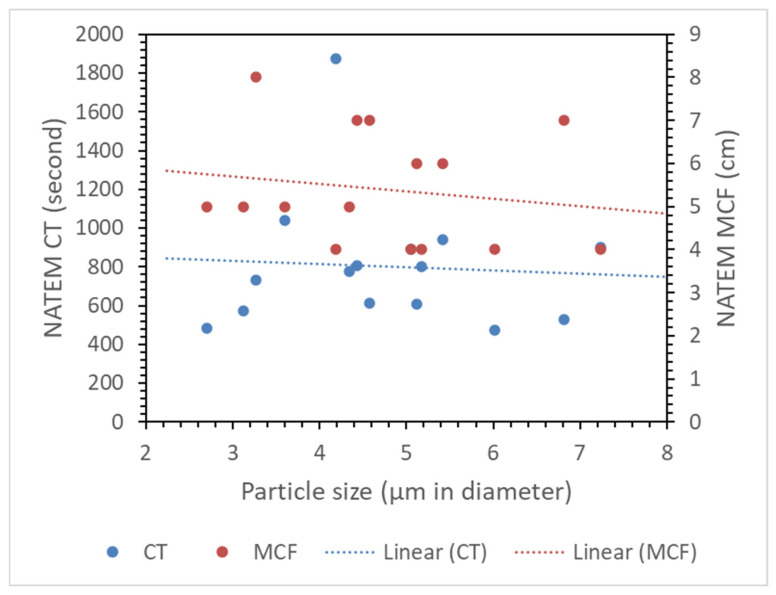
Effects of particle size on ROTEM clotting time (CT) and maximum clot firmness (MCF). ROTEM NATEM tests were performed with human plasma containing an abnormally low level of fibrinogen. It should be noted that all control particles prepared in the absence of fibrinogen resulted in non-detectable coagulation and thus were omitted. The Pearson correlation coefficient between the particle size and CT: r = −0.063 (*p* = 0.82), and MCF: r = −0.166 (*p* = 0.55).

Interestingly, the hemostatic effect of fibrinogen–CaCO_3_ particles did not correlate significantly with fibrinogen content or particle size (Figure A2 and Figure A3). ROTEM runs with an MCF lower than 4 mm are not recognized by the instrument, and any data from such runs are indicated as no detectable coagulation. Consequently, all control particles falling below this threshold were omitted for the correlation analyses, as it would be impossible to accurately assess their effects.

**Figure 4:**
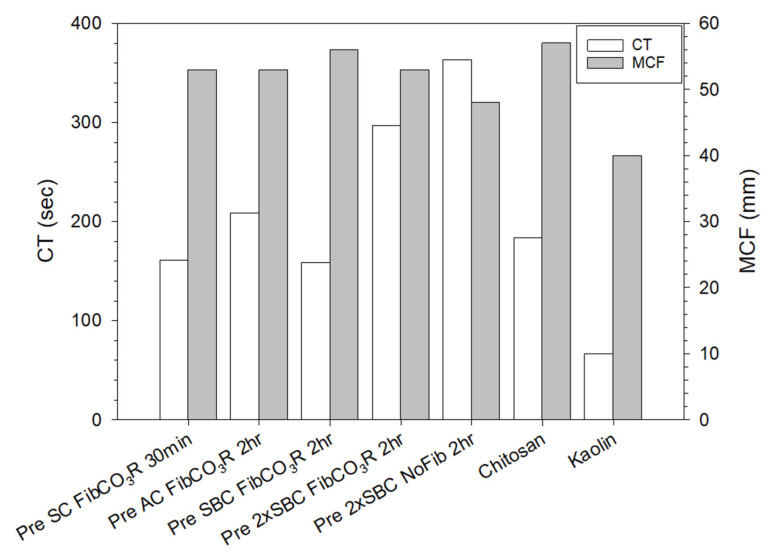
Hemostatic effects of CaCO_3_-encapsulated fibrinogen particles on human whole blood, as assessed by ROTEM. CT and MCF are shown in comparison with fibrinogen-free control particles, chitosan and kaolin.

Figure 4 illustrates the hemostatic effects of the particles on human whole blood as measured by CT and MCF. Among the tested formulations, Pre SBC FibCO_3_R 2 h exhibited the most pronounced effect, reducing CT to 159 s and increasing MCF to 56 mm, indicating rapid clot initiation and strong clot structure. Pre SC FibCO_3_R 30 min and Pre AC FibCO_3_R 2 h showed intermediate effects, with CT values of 161 and 209 s and MCF of 53 mm. Pre 2×SBC FibCO_3_R 2 h demonstrated a longer CT (297 s) and comparable MCF of 53 mm.

The fibrinogen-free control (Pre 2×SBC NoFib 2 h) exhibited the weakest performance, with the longest CT (363 s) and a lower MCF (48 mm), confirming the critical role of fibrinogen in clot initiation and strength. When compared with benchmark hemostatic agents, chitosan generated an MCF comparable to Pre SBC FibCO_3_R 2 h (57 mm) but required a longer CT (184 s), whereas kaolin produced the fastest clot initiation (CT 67 s) but the lowest MCF (40 mm). Collectively, these results indicate that these fibrinogen–CaCO_3_ particles demonstrate hemostatic performance similar to chitosan while exerting a less pronounced effect on CT than kaolin, yet offering a more substantial enhancement of MCF.

In summary, encapsulated fibrinogen is essential for hemostatic activity, and particles enhance clot strength; however, no clear relationship between fibrinogen content or particle size and ROTEM outcomes was identified (Figure A2 and Figure A3).

### 2.5. Self-Propelling Properties

As summarized in Table 2, fibrinogen-containing particles consistently showed longer lag times and faster speeds than their NoFib control particles. For instance, Pre SBC FibCO_3_R 2 h showed the longest lag time (16.612 s) with a speed of (1.664 cm/s), while Pre SBC NoFib 2 h showed the shortest lag time (0.444 s) and lowest speed (1.194 cm/s). Fibrinogen particles, such as Pre SC Fib2× 30 min, demonstrated significantly enhanced motility (4.221 cm/s) with moderate lag time (2.411 s), indicating that increased fibrinogen concentration and reduced reaction time enhance particle movement.

Among fibrinogen–CaCO_3_ particles, SC-based formulations generally outperformed SBC-based counterparts. For example, Pre SC FibCO_3_R 2 h exhibited a shorter lag time (2.744 s) and higher speed (3.261 cm/s) than Pre SBC FibCO_3_R 2 h with a much prolonged lag time (16.612 s) and reduced speed (1.664 cm/s), whereas Pre 2×SBC FibCO_3_R 2 h showed improved performance with a shorter lag time (2.511 s) and higher speed (3.154 cm/s), indicating an effect of SBC concentration.

Notably, Pre SC NoFib 30 min achieved a speed of 3.426 μm/s, suggesting that SC alone may contribute to enhanced motility even in the absence of fibrinogen. However, the inclusion of fibrinogen consistently elevated performance metrics across SC-based samples.

**Figure A4:**
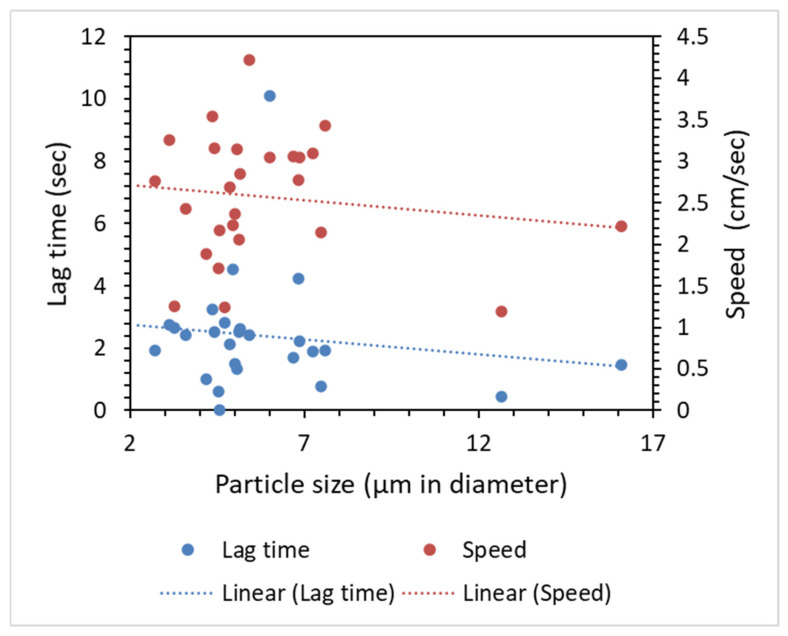
Effects of particle size on self-propelling lag time and speed. The Pearson correlation coefficient between the particle size and lag time: r = −0.138 (*p* = 0.50), and speed: r = −0.161 (*p* = 0.43).

**Figure A5:**
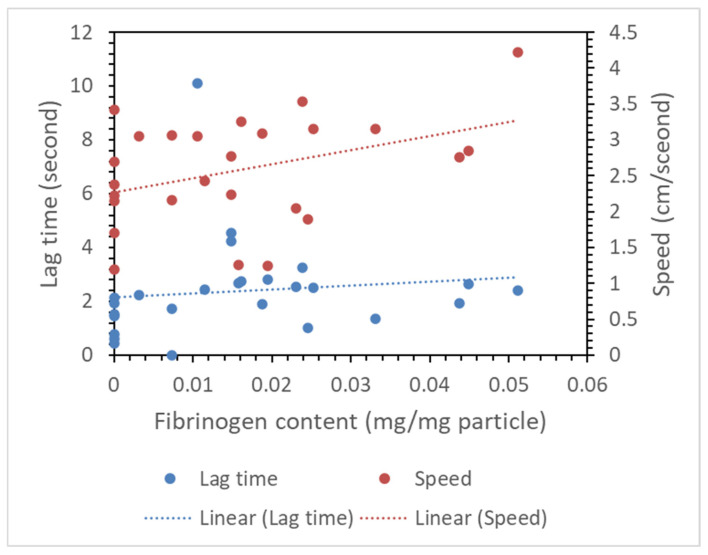
Effects of fibrinogen content on self-propelling lag time and speed. The Pearson correlation coefficient between the particle size and lag time: r = −0.053 (*p* = 0.80), and speed: r = 0.371 (*p* = 0.06).

These results demonstrate that fibrinogen inclusion, salt type and composition, and reaction time are critical determinants of activation kinetics and motility. SC-based particles, particularly those with optimized reaction times and concentrations, offer superior performance for applications requiring rapid and sustained movement. Two formulations (Pre SC Fib_low_CO_3_R 2 h and Pre SBC FibCO_3_R 2 h) exhibited unusually long lag times (>10 s) compared to others. Self-propelling properties showed no significant correlation with particle size or fibrinogen content (Figure A4 and Figure A5).

**Figure 5:**
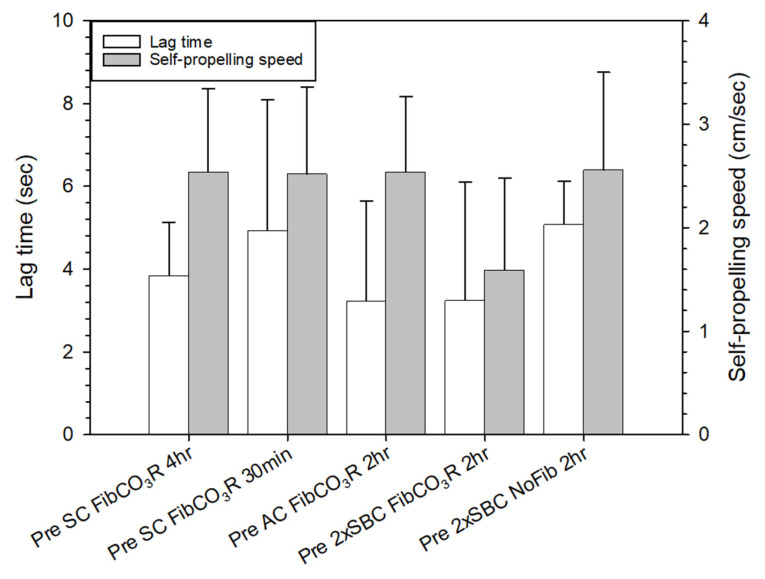
Lag time and self-propelling speed of CaCO_3_-based particle formulations measured in citrated plasma with a low fibrinogen concentration. Each value represents mean ± SD (*n* = 3). Plasma measurements reflect the impact of higher viscosity and particle–protein interactions on propulsion performance, resulting in increased lag times and reduced speeds for most formulations relative to water-based measurements.

Figure 5 compares the lag time and self-propelling speed of the different CaCO_3_-based particle formulations. Lag time ranged from approximately 3 to 5 s, while self-propelling speed varied roughly from 1.6 to 2.6 cm/s across all groups. Among the fibrinogen-containing particles, Pre AC FibCO_3_R 2 h and Pre 2×SBC FibCO_3_R 2 h exhibited the shortest lag times (~3 s), indicating faster initiation of propulsion. In contrast, Pre SC FibCO_3_R 30 min and Pre 2×SBC NoFib 2 h showed longer lag times (~4.5–5 s). Self-propelling speed was relatively consistent across formulations, with most groups achieving speeds near 2.5 cm/s, except Pre 2×SBC FibCO_3_R 2 h, which showed slightly lower speed (~1.6 cm/s). Despite these descriptive differences, no statistically significant differences were detected between groups due to the high variability within measurements. It is also worth noting that, even in citrated plasma with low-fibrinogen concentration, the Pre SBC FibCO_3_R 2 h formulation triggered rapid coagulation, which prevented completion of the propulsion test.

Lag time and self-propelling speed were compared between plasma and water for the five particle formulations (Table 2). Plasma generally increased lag time and reduced propulsion speed, with the exception of Pre AC FibCO_3_R 2 h, which showed a shorter lag in plasma. The largest lag increase occurred in Pre 2×SBC NoFib 2 h (138%), which was also the only statistically significant change (*p* = 0.009), suggesting that fibrinogen absence amplifies plasma-induced delays. Although other formulations exhibited large effect sizes, these did not reach statistical significance due to the high variability within measurements and the small sample size (*n* = 3).

In summary, self-propulsion behavior is sensitive to formulation and environment, with evidence of trade-offs between activation kinetics and speed, but relationships remain variable and require further investigation.

## 3. Discussion

CaCO_3_ microparticles are widely recognized for their biocompatibility, tunable porosity, and ability to encapsulate bioactive molecules, making them attractive for drug delivery and biomedical applications [32,33,34]. Their polymorphism—ranging from amorphous CaCO_3_ to vaterite, aragonite, and calcite—offers opportunities for functional tailoring but also introduces challenges in controlling particle morphology, stability, and protein compatibility [35,36].

This study demonstrates the successful development and characterization of fibrinogen–CaCO_3_ particles prepared via a precipitation method, with a focus on optimizing hemostatic efficacy and self-propelling behavior. The results highlight the critical influence of formulation parameters—including salt type and concentration, mixing sequence, and fibrinogen loading—on particle yield, clotting performance, and propulsion dynamics.

The carbonate source significantly influenced particle yield, morphology, and functional performance, consistent with prior reports on CaCO_3_ crystallization kinetics [37]. The highest yield obtained with SC as the carbonate source may be ascribed to its fastest reaction with CaCl_2_ compared to AC and SBC. These CaCO_3_ microcapsules likely underwent encapsulation of proteins and phase transition from vaterite to calcite in various aqueous solutions [38]. The reaction rate of CaCO_3_ with TXA^+^, which affects self-propelling and release of procoagulants, can be adjusted by controlling the crystal phase of CaCO_3_ [39].

SC has been the main carbonate source to produce CaCO_3_ with high yield and the main crystalline phase of vaterite, which is a metastable phase of crystalline CaCO_3_ [37,40]. However, SC’s high alkalinity (pH > 11) risks fibrinogen denaturation, as supported by our observation of protein precipitation at elevated pH. To mitigate this, AC and SBC were explored as alternatives, offering milder pH conditions (pH 7.8–8.2) and improved protein stability [41,42]. These findings underscore the trade-off between crystallization kinetics and protein integrity, a critical consideration for scaling up.

Furthermore, fibrinogen was dissolved in either SC or CaCl_2_ solutions with different mixing orders. Strategies involving sequential addition of carbonate to calcium (CO_3_ → CaCl_2_) or vice versa (CaCl_2_ → CO_3_) produced higher yields than simultaneous fibrinogen loading in both solutions. This supports the hypothesis that controlled nucleation and growth phases are essential for CaCO_3_ precipitation and efficient encapsulation.

Fluorescence microscopy confirmed fibrinogen encapsulation, and particle sizes were comparable to those reported for other CaCO_3_-based carriers [37]. It is hypothesized that the reaction between calcium and carbonate ions may occur around the fibrinogen macromolecule, potentially acting as a template or a nucleation point for CaCO_3_ precipitation, which results in encapsulation of fibrinogen inside. However, this mechanism warrants further investigation [43]. Additionally, these CaCO_3_ microcapsules likely underwent protein encapsulation and phase transition from vaterite to calcite in various aqueous solutions [38].

Interestingly, fibrinogen-loaded particles exhibited more amorphous morphologies than protein-free controls, suggesting mutual influence between protein conformation and CaCO_3_ crystallization [44,45]. Such interactions are complex and warrant further mechanistic studies.

The following mechanistic model can be proposed for CaCO_3_ particle formation and fibrinogen incorporation:

1. Pre-nucleation stage: Fibrinogen in solution interacts with Ca^2+^ and/or CO_3_^2−^ ions, potentially forming complexes.
2. Nucleation stage: Local supersaturation triggers formation of CaCO_3_ nuclei, which may occur either: in the bulk solution or in proximity to fibrinogen molecules.
3. Growth stage: Crystals grow through ion addition; fibrinogen at the surface may inhibit or redirect crystal growth.
4. Encapsulation stage: Protein becomes entrapped within the forming mineral matrix.

This model provides a framework for interpreting the observed trade-offs between particle yield, size, morphology and fibrinogen incorporation, but requires further validation using techniques such as in situ imaging, kinetic monitoring, or polymorph characterization. ROTEM testing confirmed that fibrinogen encapsulation is essential for clot formation. Conversely, NoFib controls showed no detectable coagulation of low-fibrinogen plasma, reinforcing the functional necessity of fibrinogen. However, no clear quantitative correlation between fibrinogen content and clotting performance was observed.

This apparent decoupling may be explained by several factors:

- Encapsulation measurements include both active and inactive fibrinogen,
- Hemostatic activity depends not only on total content but also on release kinetics and accessibility,
- CaCO_3_ dissolution under neutral ROTEM conditions may be limited, restricting fibrinogen availability.

These factors suggest that functional performance is governed by effective delivery and release rather than total loading alone.

ROTEM testing with whole blood confirmed that the CaCO_3_ particles contained functional fibrinogen and enhanced clot initiation and firmness, following trends similar to those observed in plasma. However, the hemostatic effects were less pronounced in whole blood, since non-fibrinogen controls still produced coagulation within reference ranges for whole blood (CT 254–837 s; MCF 46–69 mm).

Chitosan and kaolin, two established hemostatic agents, showed expected response patterns [46]. Chitosan, a polycationic biopolymer that agglutinates erythrocytes and interacts with platelets, produced high MCF with moderate CT, reflecting strong clot consolidation driven by electrostatic cell–polymer interactions rather than rapid coagulation factor activation [47]. In contrast, kaolin, a classical activator of FXII and the intrinsic pathway, accelerated coagulation onset (short CT) but generated lower MCF [48,49], consistent with rapid triggering of clotting without substantial enhancement of fibrin polymerization or cross-linking under the conditions tested.

In comparison, the hemostatic efficacy of fibrinogen-loaded CaCO_3_ particles combined with TXA^+^ arises from multiple complementary and synergistic mechanisms acting at several stages of hemostasis. These include localized delivery of fibrinogen to promote rapid fibrin network formation and clot reinforcement, calcium-mediated support of coagulation reactions through Ca^2+^ release from the CaCO_3_ matrix, and stabilization of the newly formed fibrin clot via antifibrinolytic inhibition by TXA^+^. In addition, transient particle transport enhances local accumulation of hemostatic components at the bleeding site. This multimodal mechanism distinguishes the system from single-component topical hemostats, while further in vivo studies are required to fully delineate their relative contributions under active bleeding conditions.

Self-propelling behavior was observed across all fibrinogen–CaCO_3_ formulations, with speeds ranging from 1.194 cm/s to 4.221 cm/s. The fastest propulsion was recorded in Pre SC Fib2× 30 min, which also had one of the shortest lag times, indicating rapid activation and strong driving force. These results suggest that increased fibrinogen concentration and reduced reaction time enhance propulsion, possibly by facilitating more vigorous CO_2_ generation during the neutralization reaction with TXA^+^. While fibrinogen inclusion generally increased lag time, this trade-off was offset by improved clotting performance, making these formulations promising for internal bleeding scenarios where rapid delivery is critical.

Importantly, propulsion performance did not show strong correlations with particle size or fibrinogen content, reflecting the complex coupling between chemical reactivity, particle morphology, and surrounding media properties. Given the high variability and small sample size, these observations should be interpreted cautiously.

Differences in propulsion between plasma and water largely reflect plasma’s higher viscosity and protein content, which slow bubble nucleation, prolong lag time, and reduce propulsion speed [50,51]. Additionally, protein adsorption onto particle surfaces may alter acid–base reaction kinetics, slowing CO_2_ generation and propulsion [52].

Across formulations, particles still achieved rapid propulsion, with speeds sufficient for movement under bleeding [21]. While propulsion was largely governed by CaCO_3_–acid reactions, fibrinogen remained critical for both propulsion and clotting outcomes. The ability to combine propulsion with intrinsic hemostatic components (e.g., fibrinogen, calcium ions) addresses a key limitation of conventional topical agents, which often fail under high-flow or non-compressible conditions.

Given the presence of multiple propulsion mechanisms and the complex rheology of blood in wounds—including turbulent or pulsatile flow, increased viscosity relative to water or plasma, and heterogeneous cellular constituents—particles were not expected to sustain comparable speeds or unidirectional motion under active bleeding [21]. These findings highlight the potential of self-propelling, protein-loaded CaCO_3_ particles as next-generation hemostatic agents for non-compressible hemorrhage, a leading cause of preventable trauma deaths. Unlike passive dressings, these particles actively navigate against blood flow, deliver procoagulants (fibrinogen, thrombin), and leverage TXA^+^ for both propulsion and antifibrinolytic protection. This multifunctionality addresses key limitations of current hemostatic technologies.

CaCO_3_ is widely employed as a delivery platform owing to its abundance, biocompatibility, and biodegradability [34]. Nonetheless, a full assessment of potential cytotoxic, immunological, and thrombogenic risks will be needed to establish the complete safety profile of this hemostatic particle.

Notably, previous studies using self-propelling CaCO_3_ systems—including thrombin-loaded and TXA^+^-modified particles—reported no adverse local or systemic effects in small or large animal hemorrhage models [21,53]. In addition, our previous studies demonstrated minimal hemolysis associated with CaCO_3_-based formulations [29]. Combined with published evidence demonstrating low cytotoxicity and negligible hemolytic activity of bioactive compound–CaCO_3_ particles [54,55], these data collectively suggest that CaCO_3_-encapsulated fibrinogen particles are likely to exhibit favorable biocompatibility.

As this study focused on the premise of making and characterizing the particles, it has limitations. One key limitation is the relatively small sample size. However, given the simplicity of the preparation process, the wide availability and low cost of raw materials, further large-scale studies are warranted. Moreover, characterization of these particles using other analytical techniques (e.g., Fourier-transform infrared spectroscopy, electron microscopy, and X-ray diffraction) would offer insights into the chemical and physical structures of the particles, the mechanistic model for particle formation and fibrinogen incorporation, facilitating further optimization. More importantly, our study was limited to in vitro models and simplified propulsion assays, which do not fully replicate the complexity of active bleeding. Future work should include:

1. In vivo validation in animal models of non-compressible hemorrhage to assess efficacy, safety, and systemic coagulation effects, including direct comparison with established hemostatic standards such as chitosan-based Celox granules and kaolin-impregnated Combat Gauze.
2. Mechanistic studies on fibrinogen release kinetics, dosing effects, particle–blood interactions, and the role of carbonate polymorphs in propulsion and clotting.
3. Integration of additional bioactives (e.g., thrombin, alginate, antimicrobial peptides) for multifunctional wound care.
4. Optimization of particle architecture (e.g., porosity, surface roughness) to fine-tune propulsion and release profiles.
5. Safety profiling, including thrombosis risk and biodisposition, to ensure clinical translation.

## 4. Materials and Methods

### 4.1. Materials

Ammonium carbonate (AC, ACS grade), sodium carbonate (SC, 98% purity), sodium bicarbonate (SBC, >99% purity), anhydrous calcium chloride (CaCl_2_, ≥96% purity), 4-(2-hydroxyethyl)-1-piperazineethanesulfonic acid (HEPES, 99% purity), tranexamic acid (>98% purity), fluorescein isothiocyanate (FITC), Invitrogen™ Novex™ tris-glycine mini protein gels (8–16%, 1.0 mm, WedgeWell™ format), iBright™ prestained protein ladder, sodium dodecyl sulphate (SDS) buffer, dithiothreitol (DTT, 99.5% purity), and coomassie blue (SimplyBlue™ SafeStain) were purchased from Fisher Scientific (Ottawa, ON, Canada). Chitosan powder (85% deacetylation, medium molecular weight; viscosity 0.34 Pa·s for 1% solution in 1% acetic acid) and kaolin powder (USP grade) were obtained from Sigma-Aldrich Canada Ltd. (Oakville, ON, Canada). Fibrinogen concentrate was sourced from CSL Behring (King of Prussia, PA, USA), bovine thrombin (129 U/mg) from VWR International (Mississauga, ON, Canada), citrated human plasma with abnormally low fibrinogen (<0.7 g/L) from Precision BioLogic Inc. (Dartmouth, NS, Canada), and normal human whole blood from Fisher Scientific (Ottawa, ON, Canada).

### 4.2. Preparation of Self-Propelling Particles

Self-propelling particles were composed of fibrinogen–CaCO_3_ microparticles and protonated TXA (TXA^+^). Fibrinogen–CaCO_3_ particles were synthesized via a precipitation method as detailed below.

#### 4.2.1. Precipitation Method

The precipitation method was adapted from previously validated protocols [22]. In a typical preparation, 11.9 mg HEPES and 100 mg fibrinogen were dissolved in 5 mL Milli-Q water. This solution was slowly mixed with 174.9 mg SC to avoid premature fibrinogen precipitation. Separately, 183.1 mg CaCl_2_ was dissolved in 5 mL Milli-Q water and gradually added to the carbonate solution under stirring (200 rotations per minute (RPM), room temperature, 120 min), resulting in the formation of fibrinogen–CaCO_3_ particles. Resulting particles were collected by centrifugation (3000 RPM, 10 min), washed thrice with Milli-Q water, and lyophilized.

Several formulations were prepared in 3 independent batches, performed on separate days using freshly prepared reagents. All characterization and functional assays were conducted on independently prepared batches unless otherwise specified.

Particle yield (%) was defined as:

$$Yield=\;\frac{mass\;of\;dried\;particles\;recovered}{theoretical\;mass\;of\;{CaCO}_{3}\;formed+initial\;fibrinogen\;input}\;\times 100$$

The theoretical CaCO_3_ mass was calculated based on limiting reagent stoichiometry.

Variations included adjusting carbonate source (SC, SBC, AC) and concentration, fibrinogen concentration, mixing order and speed, and reaction time. Control particles (without fibrinogen) were prepared under identical conditions. Table 1 summarizes the preparation conditions and yields for various batches.

#### 4.2.2. Protonation of TXA

TXA^+^ was prepared by dissolving TXA (10% *w*/*v*) in Milli-Q water and adjusting the pH to 4.3 using HCl, as previously described [21]. The solution was lyophilized to obtain solid TXA^+^.

### 4.3. Characterization of Particles

The morphologies of CaCO_3_-encapsulated fibrinogen particles were characterized by light and fluorescent microscopy. As essential requirements for their use in non-compressible hemorrhage control, the hemostatic and self-propelling properties of the particles were measured by a number of methods as described below. The presence and hemostatic effects of fibrinogen were quantified by gel electrophoresis and rotational thromboelastometry (ROTEM), respectively. Both methods have been used to analyze fibrinogen [56]. Self-propelling ability of the particle when mixed with TXA^+^ in water was measured following the method as previously reported [22]. Furthermore, self-propulsion phenomena of the particles were video recorded in real time and quantitatively analyzed for their response time and moving speed.

#### 4.3.1. Light and Fluorescent Microscopy

Microscopy images were acquired by ZEISS LSM 800—Airyscan, monitored by ZEN BLUE (Carl Zeiss Canada Ltd., North York, ON, Canada) for all the particles. A small sample of particles was spread onto a glass slide, and then shaken to remove excess particles.

##### FITC Labelling

FITC-labeled fibrinogen was prepared by reacting FITC with fibrinogen (0.15 molar ratio) in dimethylformamide, incubated in the dark for 30 min at 200 RPM. FITC-labeled particles were washed with isopropanol to remove excess dye. Controls included FITC-labeled particles without fibrinogen.

##### Imaging Analysis

Fluorescence was excited at 488 nm and detected across 488–620 nm. All imaging parameters were kept constant across all samples to ensure comparability. Imaging settings were optimized empirically and fixed as follows:

- Laser intensity: 2%
- Pinhole: 43 μm (1 Airy Unit)
- Master gain: 650 V
- Digital offset: −15,000
- Digital gain: 1.0

Fluorescence intensity values are reported in arbitrary units (0–65,535) determined by detector output. While not absolute measurements, they are internally consistent under fixed acquisition parameters, enabling valid comparison between samples.

All images were analyzed using ZEISS ZEN software (version 3.1). The HISTO function was used to select regions corresponding to individual particles and to calculate their average fluorescence intensity. Background fluorescence was determined using NoFib-FITC control samples (particles without fibrinogen), which account for signal arising from entrapped free FITC independent of fibrinogen presence. These background values were subtracted from the fluorescence intensities of fibrinogen-loaded particles using the Image Calculator function. The resulting corrected intensities were assumed to represent fluorescence specifically associated with the encapsulated fibrinogen.

#### 4.3.2. Gel Electrophoresis

To quantify fibrinogen encapsulation, 10 mg particles were dissolved in 0.5 mL TXA^+^ (pH 4.3) for 2 h. Supernatants were mixed with SDS buffer, DTT, and Milli-Q water, heated at 90 °C for 5 min, and analyzed via SDS-PAGE (125 V, 50 mA, 100 min). Gels were stained with Coomassie Blue and compared to molecular weight standards.

The relative optical intensity of each band was quantified by densitometric analysis using the program ImageJ (version 1.54j) downloaded from http://rsb.info.nih.gov/ij/index.html (accessed on 15 June 2024). The fibrinogen content in the CaCO_3_ particle samples was estimated by comparing the band intensity (peak area) to a standard curve generated from known fibrinogen concentrations ranging from 0.04 to 1 mg/mL included in each run.

#### 4.3.3. Rotational Thromboelastometry (ROTEM)

ROTEM was used to assess the hemostatic effects of the particles, following established protocols [57]. Tests were performed at 37 °C on a ROTEM Delta analyzer (Instrumentation Laboratory, Bedford, MA, USA) using human plasma with a low concentration of fibrinogen and normal whole blood. For each run, 6 mg of fibrinogen-loaded CaCO_3_ particles, or blank CaCO_3_ controls, were added to a ROTEM cup, followed by 20 µL of star-tem (0.2 M CaCl_2_) and 300 µL of citrated plasma or whole blood, as per the manufacturer’s NATEM procedure. All measurements were recorded for at least 60 min. The primary ROTEM parameters evaluated were clotting time (CT) and maximum clot firmness (MCF).

#### 4.3.4. Self-Propulsion Test

Self-propulsion was assessed by mixing fibrinogen-loaded or unloaded CaCO_3_ particles with TXA^+^ at a mass ratio of 1.2, as previously reported [22]. The mixture was placed on an aluminum weigh boat and aliquoted into approximately equal piles. A sealed pipette containing 0.6 mL of water or citrated plasma with abnormally low fibrinogen was applied to the sample, initiating the reaction. Particle movement was tracked using Tracker software (version 6.2) [https://physlets.org/tracker/] (accessed on 15 June 2024). Metrics included lag time (time to movement initiation) and speed (cm/s over the first 20 data points).

#### 4.3.5. Statistical Analysis

Data are presented as mean ± standard deviation (SD), with *n* = 3 unless otherwise stated. Normality and homogeneity of variance were assessed using Shapiro–Wilk and Levene’s tests, respectively. Parametric data that satisfied both assumptions (particle size, self-propulsion lag time and speed) were analyzed using one-way ANOVA and *t*-tests. All statistical analyses were conducted using SPSS Statistics 28 (IBM Corporation, Armonk, NY, USA). A *p* value of less than 0.05 was considered significant.

## 5. Conclusions

This study demonstrates the feasibility of precipitation-based encapsulation of fibrinogen in CaCO_3_ microparticles. Fibrinogen incorporation was essential for hemostatic activity, as fibrinogen-free particles showed no detectable clot formation. Formulation parameters, including carbonate source, concentration, reaction time, and mixing sequence, strongly influenced particle yield, fibrinogen loading, morphology, and propulsion behavior.

The results highlight clear formulation-dependent trade-offs, with no single condition optimizing all performance metrics simultaneously. Overall, these findings establish a proof-of-concept system and underscore the need for further studies to evaluate performance under physiologically relevant conditions and to assess translational potential.

## Figures and Tables

**Table 1:** Preparation of CaCO_3_-encapsulated fibrinogen particles by the precipitation method under various conditions.

Batch ^1^	Carbonate Concentration (M)	Fibrinogen Concentration (g/L)	Calcium Concentration (M)	Mixing Speed (RPM)	Mixing Time (min)	Mixing Step	Yield (%) ^2^
Pre SC 2×CaCl_2_ FibCO_3_R 2 h	0.33	20	0.66	200	120	CaCl_2_ -> FibCO_3_	66
Pre SC 0.5×CaCl_2_ FibCO_3_R 2 h	0.33	20	0.165	200	120	CaCl_2_ -> FibCO_3_	39
Pre SC Fib2× 2 h	0.33	20 × 2	0.33	200	120	FibCaCl_2_ -> FibCO_3_	37
Pre SC Fib2× 30 min	0.33	20 × 2	0.33	200	30	FibCaCl_2_ -> FibCO_3_	36
Pre SC NoFib 30 min ^3^	0.33	0	0.33	200	30	CaCl_2_ -> CO_3_	83 ± 1
Pre SC FibCO_3_R 4 h	0.33	20	0.33	200	240	CaCl_2_ -> FibCO_3_	58
Pre SC FibCO_3_R 2 h	0.33	20	0.33	200	120	CaCl_2_ -> FibCO_3_	48 ± 2
Pre SC Fib_low_CO_3_R 2 h	0.33	15	0.33	200	120	CaCl_2_ -> FibCO_3_	64
Pre SC FibCO_3_R 30 min	0.33	20	0.33	200	30	CaCl_2_ -> FibCO_3_	54
Pre SC FibCO_3_ 30 min	0.33	20	0.33	200	30	FibCO_3_ -> CaCl_2_	55
Pre SC FibCaCl_2_R 30 min	0.33	20	0.33	200	30	FibCaCl_2_ -> CO_3_	47
Pre SC FibCaCl_2_ 30 min	0.33	20	0.33	200	30	CO_3_ -> FibCaCl_2_	54
Pre 2×SC 2×CaCl_2_ FibCaCl_2_ 30 min	0.66	20	0.66	200	30	CO_3_ -> FibCaCl_2_	73
Pre 2×SC 2×CaCl_2_ NoFib 30 min ^3^	0.66	0	0.66	200	30	FibCaCl_2_ -> CO_3_	85
Pre 0.5×SC 0.5×CaCl_2_ FibCO_3_ 30 min	0.165	20	0.165	200	30	FibCO_3_ -> CaCl_2_	29
Pre 0.5×SC 0.5×CaCl_2_ NoFib 30 min ^3^	0.165	0	0.165	200	30	CO_3_ -> CaCl_2_	69
Pre SC FibCO_3_R 2×Spd 30 min	0.33	20	0.33	400	30	CaCl_2_ -> FibCO_3_	55
Pre SC FibCO_3_R 2×Spd 4 h	0.33	20	0.33	400	240	CaCl_2_ -> FibCO_3_	47
Pre SC NoFib 2×Spd 4 h ^3^	0.33	0	0.33	400	240	CaCl_2_ -> CO_3_	78
Pre AC FibCO_3_R 2 h	0.33 M AC	20	0.33	200	120	CaCl_2_ -> FibCO_3_	33 ± 4
Pre AC NoFib 2 h ^3^	0.33 M AC	0	0.33	200	120	CaCl_2_ -> CO_3_	35
Pre AC Fib2× 2 h	0.33	20 × 2	0.33	200	120	FibCaCl_2_ -> FibCO_3_	19
Pre AC Fib2× 30 min	0.33	20 × 2	0.33	200	30	FibCaCl_2_ -> FibCO_3_	11
Pre SBC FibCO_3_R 2 h	0.33	20	0.33	200	120	CaCl_2_ -> FibCO_3_	30 ± 5
Pre SBC NoFib 2 h ^3^	0.33	0	0.33	200	120	CaCl_2_ -> CO_3_	41
Pre 2×SBC FibCO_3_R 2 h	0.66	20	0.33	200	120	CaCl_2_ -> FibCO_3_	56 ± 7
Pre 2×SBC NoFib 2 h ^3^	0.66	0	0.33	200	120	CaCl_2_ -> CO_3_	81 ± 1

^1^ Each batch was named as Pre for Precipitation, carbonate type (AC for ammonium carbonate, SBC for sodium bicarbonate, SC for sodium carbonate) with changes to the default concentrations of carbonate and calcium solutions (e.g., 2×SC 2×CaCl_2_), the solution containing fibrinogen (FibCO_3_ for the carbonate solution containing fibrinogen, FibCaCl_2_ for the CaCl_2_ solution containing fibrinogen, Fib2× for both carbonate and CaCl_2_ solutions containing fibrinogen), mixing speed if doubled (2×Spd), reaction time, R for reversed order of mixing step (i.e., CaCl_2_ solution was added into carbonate solution CaCl_2_ -> FibCO_3_ instead of the default procedure FibCO_3_ -> CaCl_2_); ^2^ calculated as the actual amount of product divided by the sum of theoretical amount of CaCO_3_ that should be produced and initial amount of added fibrinogen in the preparation; ^3^ control particles, i.e., CaCO_3_ particles in the absence of fibrinogen.

**Table 2:** The size, fibrinogen content, hemostatic and self-propelling properties of fibrinogen–CaCO_3_ particles prepared by the precipitation method under different conditions.

Sample ID	Particle Size in Diameter (µm, *n* = 10)	Encapsulated Fibrinogen mg/mg Particle	ROTEM		Lag Time (s)	Self-Propelling Speed (cm/s)
			CT (s)	MCF (mm)		
Pre SC 2×CaCl_2_ FibCO_3_R 2 h	3.602 ± 0.307	0.0115	1039	5	2.433 ± 0.705	2.431 ± 0.513
Pre SC 0.5×CaCl_2_ FibCO_3_R 2 h	6.856 ± 0.651	0.0031	No detectable coagulation		2.221 ± 0.310	3.049 ± 0.685
Pre SC Fib2× 2 h	5.115 ± 0.537	0.0231	609	6	2.532 ± 0.833	2.052 ± 0.368
Pre SC Fib2× 30 min	5.412 ± 0.574	0.0512	940	6	2.411 ± 0.724	4.221 ± 0.803
Pre SC NoFib 30 min	7.593 ± 0.549	0	No detectable coagulation		1.922 ± 0.379	3.426 ± 0.766
Pre SC FibCO_3_R 4 h	4.347 ± 0.548	0.0239	776	5	3.256 ± 0.907	3.541 ± 0.741
Pre SC FibCO_3_R 2 h	3.117 ± 0.274	0.0161	444 ± 185	4.5 ± 0.7	2.744 ± 0.769	3.261 ± 0.892
Pre SC Fib_low_CO_3_R 2 h	6.019 ± 1.148	0.0105	473	4	10.116 ± 4.273	3.048 ± 1.323
Pre SC FibCO_3_R 30 min	5.168 ± 0.782	0.0450	604	4	2.633 ± 0.434	2.852 ± 0.678
Pre SC FibCO_3_ 30 min	5.634 ± 0.655	0.0187	900	4	1.889 ± 0.635	3.094 ± 0.674
Pre SC FibCaCl_2_R 30 min	4.946 ± 0.625	0.0149	800	4	4.537 ± 4.047	2.237 ± 1.397
Pre SC FibCaCl_2_ 30 min	4.185 ± 0.484	0.0246	1874	4	1.001 ± 0.802	1.888 ± 0.819
Pre 2×SC 2×CaCl_2_ FibCaCl_2_ 30 min	5.056 ± 0.596	0.0331	889	4	1.344 ± 0.351	3.149 ± 0.563
Pre 2×SC 2×Cl NoFib 30 min	4.532 ± 0.455	0	No detectable coagulation		0.622 ± 0.417	1.707 ± 0.591
Pre 0.5×SC 0.5×CaCl_2_ FibCO_3_ 30 min	4.716 ± 1.148	0.0195	-	-	2.813 ± 3.083	1.246 ± 0.892
Pre 0.5×SC 0.5×Cl NoFib 30 min	7.471 ± 0.485	0	No detectable coagulation		0.767 ± 0.133	2.149 ± 1.101
Pre SC FibCO_3_R 2×Spd 30 min	2.701 ± 0.153	0.0437	482	5	1.922 ± 0.317	2.759 ± 0.644
Pre SC FibCO_3_R 2×Spd 4 h	6.684 ± 0.781	0.0073	-	-	1.711 ± 0.395	3.061 ± 0.653
Pre SC NoFib 2×Spd 4 h	5.003 ± 0.711	0	No detectable coagulation		1.511 ± 0.383	2.371 ± 0.887
Pre AC FibCO_3_R 2 h	6.818 ± 1.772	0.0148	632 ± 174	6.7 ± 2.1	4.220 ± 0.966	2.776 ± 1.197
Pre AC NoFib 2 h	16.095 ± 3.865	0	No detectable coagulation		1.468 ± 0.934	2.221 ± 0.788
Pre AC Fib2× 2 h	3.262 ± 0.503	0.0158	730	8	2.668 ± 4.062	1.252 ± 0.605
Pre SBC FibCO_3_R 2 h	4.579 ± 0.975	0.0073	579 ± 45	6.5 ± 0.7	16.612 ± 7.983	1.664 ± 0.990
Pre SBC NoFib 2 h	12.657 ± 2.577	0	No detectable coagulation		0.444 ± 0.317	1.194 ± 0.589
Pre 2×SBC FibCO_3_R 2 h	4.431 ± 0.285	0.0253	657 ± 239	7.0 ± 1	2.511 ± 0.847	3.154 ± 0.728
Pre 2×SBC NoFib 2 h	4.874 ± 1.241	0	No detectable coagulation		2.133 ± 0.233	2.691 ± 0.939

## Data Availability

The original contributions presented in this study are included in the article. Further inquiries can be directed to the corresponding author.

## Abbreviations

The following abbreviations are used in this manuscript:

AC	Ammonium Carbonate
CT	Clotting Time
DTT	Dithiothreitol
FITC	Fluorescein Isothiocyanate
HEPE	4-(2-Hydroxyethyl)-1-Piperazineethanesulfonic Acid
MCF	Maximum Clot Firmness
ROTEM	Rotational Thromboelastometry
RPM	Rotations Per Minute
SBC	Sodium Bicarbonate
SC	Sodium Carbonate
SD	Standard Deviation
SDS	Sodium Dodecyl Sulphate
TXA^+^	Protonated Tranexamic Acid
